# Molecular evidence for the occurrence of Japanese encephalitis virus genotype I and III infection associated with acute Encephalitis in Patients of West Bengal, India, 2010

**DOI:** 10.1186/1743-422X-9-271

**Published:** 2012-11-15

**Authors:** Arindam Sarkar, Debjani Taraphdar, Subhra Kanti Mukhopadhyay, Sekhar Chakrabarti, Shyamalendu Chatterjee

**Affiliations:** 1 ICMR virus unit, ID & BG Hospital, 57, Dr. S. C. Banerjee Road, Beliaghata, Kolkata-700010, West Bengal, India; 2 Department of Microbiology, The University of Burdwan Golapbag, Burdwan, West Bengal, India

**Keywords:** Acute encephalitis syndrome, Japanese encephalitis virus, Genotype I, Genotype III, West Bengal

## Abstract

**Background:**

Japanese encephalitis virus (JEV), a mosquito-borne zoonotic pathogen, is the sole etiologic agent of Japanese Encephalitis (JE); a neurotropic killer disease which is one of the major causes of viral encephalitis worldwide with prime public health concern. JE was first reported in the state of West Bengal, India in 1973. Since then it is being reported every year from different districts of the state, though the vaccination has already been done. Therefore, it indicates that there might be either partial coverage of the vaccine or the emergence of mutated/new strain of JEV. Considering this fact, to understand the JEV genotype distribution, we conducted a molecular epidemiological study on a total of 135 serum/cerebrospinal fluid (CSF) samples referred and/or collected from the clinically suspected patients with Acute encephalitis syndrome (AES), admitted in different district hospitals of West Bengal, India, 2010.

**Findings:**

JEV etiology was confirmed in 36/135 (26.6%) and 13/61 (21.3%) 2–15 days’ febrile illness samples from AES cases by analyzing Mac-ELISA followed by RT-PCR test respectively. Phylogenetic analysis based on complete envelope gene sequences of 13 isolates showed the emergence of JEV genotype I (GI), co-circulating with genotype III (GIII).

**Conclusion:**

This study represents the first report of JEV GI with GIII, co-circulating in West Bengal. The efficacy of the vaccine (derived from JEV GIII strain SA-14-14-2) to protect against emerging JEV GI needs careful evaluation. In future, JE outbreak is quite likely in the state, if this vaccine fails to protect sufficiently against GI of JEV.

## Background

The mosquito-borne Japanese encephalitis virus (JEV) is an enveloped, positive-sense single-stranded RNA virus, member of the genus Flavivirus under the family Flaviviridae [1]. JEV is the sole etiologic agent of Japanese Encephalitis (JE); a neurotropic killer disease being one of the major causes of viral encephalitis in human. Since the isolation of this virus in Japan in 1935 [2], it has spread worldwide becoming a major public health problem. Worldwide case-fatality rate of JE was recorded to be 30% approximately with 30-50% of survivors developing permanent neurologic deficit/sequelae [3].

Recent studies have shown that the envelope (E) gene is an established phylogenetic marker for JEV, since this region is free from selective pressure that supports obscure long-term evolutionary relationship [4]. Altogether 5 distinct genotypes have been identified among the JEV strains [5] of which genotype III (GIII) is mostly circulated in the Southeast Asian countries, including Japan, South Korea, China, Taiwan, Vietnam, Philippines, and India [6]. However, it was recently documented that GIII is replaced by genotype I (GI) in South Korea, Thailand and China [7]. Though GIII is predominant in India, GI has been introduced in the country recently [7]. In 1973 JE outbreak was first recorded in the districts of Burdwan and Bankura in West Bengal where 700 cases and 300 deaths were reported [8,9]. Thereafter, several JE outbreaks took place in the state [10-12]. Every year sporadic JE cases are being reported indicating its endemicity in this state despite the vaccination programme undertaken by the State Health Department, Government of West Bengal [13]. In addition, the geographic features, environmental factors and socio-economic status of this state also favor JEV transmission [14]. Moreover, the reports of JE incidences in the state are the indications of either partial coverage of the vaccine or the emergence of mutated/new strain of JEV. Genetic variation of JEV circulating in West Bengal has not yet been investigated and hence to ascertain the same a molecular epidemiological study was undertaken.

## Materials and Methods

**Figure 1:**
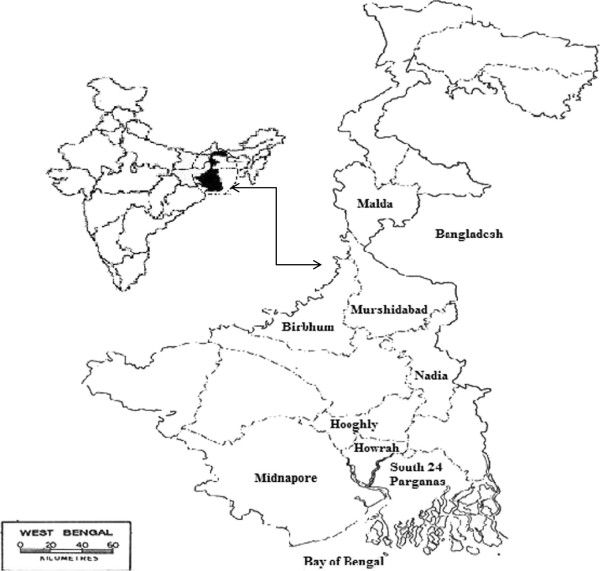
Map of West Bengal showing the location of sample collection areas.

A total of 92 serum and 43 cerebrospinal fluid (CSF) samples were referred and/or collected from 135 clinically suspected pediatric-adolescent (0–20 years old) and adult (≥ 21 years old) individuals with Acute encephalitis syndrome (AES), showing high grade fever (≥39°c) for 2–15 days including any two of the following symptoms, viz. headache, vomiting, stupor, delirium, abnormal movements, presence of kernig’s sign, convulsions, neck rigidity, altered sensorium, unconsciousness admitted in 8 different district hospitals, West Bengal during the period from July to December in 2010 (Figure 1).

All the samples were tested for IgM antibody against JEV by using IgM antibody-capture (Mac) ELISA kit (National institute of virology, Pune, India), according to the manufacturer’s protocol.

Only 61 JEV IgM negative samples with a history of ≤ 3 days’ illness were screened and 200 μl of each of them were used for virus isolation on C6/36 cell line according to the standard protocol [13]. The tissue culture fluids were collected from the samples producing prominent cytopathic effect (CPE) and subjected for RNA extraction by QIAamp RNA viral kit (Qiagen, GmbH, Hilden, Germany), following the manufacturer’s protocol.

To identify the isolates as JEV, reverse transcription-PCR (RT-PCR) was carried out with the extracted RNA (50 pg to 1μg) by Qiagen one step RT-PCR kit (Qiagen, GmbH, Hilden**,** Germany), in accordance with the manufacture’s specifications, using 0.6 μM of primer pairs [13] that specific for structural E gene sequence of JEV. The PCR products were separated by electrophoresis on 1% agarose gel, stained with ethidium bromide.

RT-PCR amplicons were purified using the Qiagen gel extraction kit (Qiagen, GmbH, Hilden**,** Germany), according to the manufacturer’s protocol, followed by direct sequencing using the BigDye Terminator Cycle Sequencing Ready Reaction Kit (Applied Biosystems, Foster City, CA, USA), as per the manufacturer’s instructions and the products were analyzed using an automated DNA sequencer, 3130XL Genetic Analyzer (PE Applied Biosystems, Foster city, CA, USA). The 1,500 nucleotides generated complete sequences of the JEV E gene that were edited and corrected using the Finch TV software (http://www.geospiza.com). Multiple sequence alignment and phylogenetic analysis were performed by using CLUSTALW (http://www.ebi.ac.uk/Tools/clustalw2/index.html) and MEGA version 5.0 software (http://www.megasofteware. net). The phylogenetic tree was constructed by the neighbor-joining method, tested with Kimura 2-parameter model.

## Results

Out of 135 samples, only 36 (26.6%) samples were reactive to JEV specific IgM antibody, of which 23 (63.8%) and 13 (36.1%) samples were CSF and serum respectively. Only 61 of the remaining 99 JEV IgM negative samples having the history of ≤ 3 days of febrile illness were selected and subsequently subjected to tissue culture resulting in 19 samples producing prominent CPE of which only 13 (21.3%) samples were identified as JE positive by RT-PCR method, consisting of 8 (61.5%) from CSF and 5 (38.4%) from serum.

We have a total of 49 (36 IgM + 13 RT-PCR positive) JE cases (36.2%) of which 30 (19 IgM + 11 RT-PCR positive) were pediatric-adolescent (61.2%) and remaining 19 (17 IgM + 2 RT-PCR positive) were found to be adult cases (38.7%). Moreover, the occurrence of JEV infection was recorded during the month of July to December with the maximum number of cases (46.9%) observed in the month of September.

**Figure 2:**
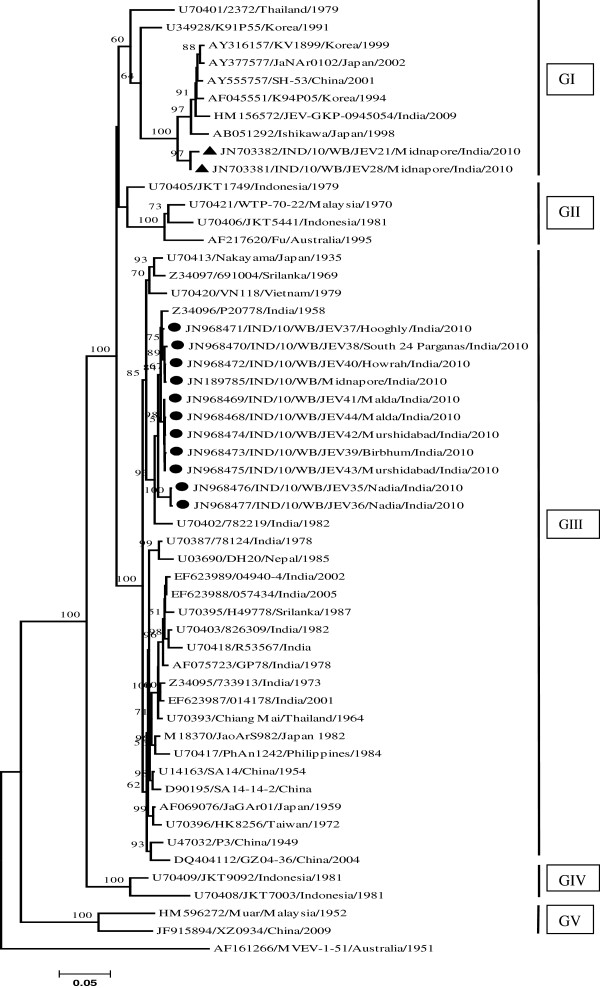
**Phylogenetic analysis of 13 JEV isolates in serum/CSF samples from AES patients, West Bengal.** The Neighbor-Joining (NJ) Phylogenetic tree based on complete envelope (E) gene nucleotide sequences of Japanese encephalitis virus (JEV) isolates/strains. The Murray valley encephalitis virus strain (MVEV-1-51) was used as an out group for generating the rooted tree. The robustness of dendrogram was evaluated by 1000 bootstrap pseudo replicates. Bootstrap values (≥50% of replicates) were shown in corresponding nodes. Horizontal branch lengths are proportional to genetic distance and vertical branch lengths have no significance. Each taxon is named systematically by mentioning the accession number, strain name, country of origin and year of isolation. The isolates’ sequences used in this study were marked with filled circle and triangle symbols. Genotypes are indicated on the right. Scale bar indicates nucleotide substitutions per site.

The Figure 2 shows the phylogenetic tree derived from 13 E gene sequences of JEV isolates along with 41 previously published JEV strains, including 12 from India and 29 from worldwide (Table 1). Dendrogram showed 2 E gene sequences of the isolates (GenBank: JN703381, JN703382) belonging to GI and comprising 89%-91% nucleotide (nt) identity with 11 E gene sequences of other isolates (GenBank: JN968468- JN968477, JN189785) belonging to GIII (Figure 2). Moreover, these 2 GI E gene sequences showed 99% nt similarity with each other and were most similar (96%) with Japanese GI strain Ishikawa (GenBank: AB051292), followed by 94%-95% nt similarity with Indian isolate JEV-GKP-0945054 (GenBank: HM156572). Eleven GIII E gene sequences showed 97%-99% nt similarity with each other and 93%-98% nt similarity with other Indian GIII strains, having the highest similarity (97%-98%) with Indian P20778 strain (GenBank: Z34096).

**Table 1:** Background information of selected strains/isolates of JEV referenced in this study

**Strains/isolates**	**Country and Year of isolation**	**Source**	**Genotype**	**GenBank Accession no.**
Nakayama	Japan, 1935	Human brain	III	U70413
JaGAr01	Japan, 1959	Mosquito	III	AF069076
JaoArS982	Japan, 1982	Mosquito	III	M18370
Ishikawa	Japan, 1998	Mosquito	I	AB051292
JaNAr0102	Japan, 2002	Mosquito	I	AY377577
Fu	Australia,1995	Human	II	AF217620
WTP-70-22	Malaysia ,1970	Mosquito	II	U70421
PhAn1242	Philipines,1984	Pig serum	III	U70417
Muar	Malaysia, 1952	Human	V	HM596272
691004	Srilanka, 1969	Human	III	Z34097
H49778	Srilanka, 1987	Human	III	U70395
DH20	Nepal, 1985	Human	III	U03690
VN118	Vietnam, 1979	Mosquito	III	U70420
K94P05	Korea, 1994	Mosquito	I	AF045551
KV1899	Korea, 1999	Pig	I	AY316157
K91P55	Korea, 1991	Mosquito	I	U34928
GP78	India, 1978	Human	III	AF075723
733913	India, 1973	Human brain	III	Z34095
P20778	India, 1958	Human	III	Z34096
R53567	India, unavailable	unavailable	III	U70418
782219	India, 1982	Human	III	U70402
826309	India, 1982	Human brain	III	U70403
R53567	India, unavailable	unavailable	III	U70418
014178	India, 2001	Human blood	III	EF623987
04940-4	India, 2002	Mosquito	III	EF623989
057434	India, 2005	Human blood	III	EF623988
78124	India, 1978	Human	III	U70387
JEV-GKP-0945054	India, 2009	Human CSF	I	HM156572
P3	China, 1949	Mosquito	III	U47032
SA14	China, 1958	Mosquito	III	U14163
SA14-14-2	China, unavailable	Vaccine	III	D90195
GZ04-36	China, 2004	Mosquito	III	DQ404112
XZ0934	China, 2009	Mosquito	V	JF915894
SH-53	China, 2001	Mosquito	I	AY555757
JKT1749	Indonesia, 1979	Mosquito	II	U70405
JKT9092	Indonesia, 1981	Mosquito	IV	U70409
JKT7003	Indonesia, 1981	Mosquito	IV	U70408
JKT5441	Indonesia, 1981	Mosquito	II	U70406
2372	Thailand, 1979	Human	I	U70401
Chiang Mai	Thailand, 1964	Human	III	U70393
HK8256	Taiwan, 1972	Mosquito	III	U03691
IND/10/WB	India, 2010	Human CSF	III	JN189785
IND/10/WB/JEV28	Midnapore, India, 2010	Human CSF	I	JN703381
IND/10/WB/JEV21	Midnapore, India, 2010	Human CSF	I	JN703382
IND/10/WB/JEV38	South 24 Pgs, West Bengal, India, 2010	Human CSF	III	JN968470
IND/10/WB/JEV37	Hooghly, West Bengal, India, 2010	Human serum	III	JN968471
IND/10/WB/JEV44	Malda, West Bengal, India, 2010	Human CSF	III	JN968468
IND/10/WB/JEV41	Malda, West Bengal, India, 2010	Human CSF	III	JN968469
IND/10/WB/JEV40	Howrah, West Bengal, India, 2010	Human CSF	III	JN968472
IND/10/WB/JEV39	Birbhum, West Bengal, India, 2010	Human CSF	III	JN968473
IND/10/WB/JEV42	Murshidabad, West Bengal, India, 2010	Human serum	III	JN968474
IND/10/WB/JEV43	Murshidabad, West Bengal, India, 2010	Human serum	III	JN968475
IND/10/WB/JEV35	Nadia, West Bengal, India, 2010	Human serum	III	JN968476
IND/10/WB/JEV36	Nadia, West Bengal, India, 2010	Human serum	III	JN968477
MVEV-1-51	Australia, 1951	Human		AF161266

All strains/isolates are JEV, except Murray valley encephalitis virus strain (MVE-1-51) used as out group for phylogenetic analysis in this study.

## Discussion

JEV infection is considered as a prime issue on public health concern in West Bengal. The present study reveals that 36/135 (26.6%) and 13/61 (21.3%) samples were positive to JE by Mac-ELISA and RT-PCR method respectively. This observation is the proof of JEV infection in recent time and to detect the total number of JE cases, ELISA negative acute samples (from ≤ 3 days’ febrile illness) should be confirmed by RT-PCR test.

JE incidences (61.2% vs. 38.7%) were higher in pediatric-adolescent age group than adult because pediatrics were infected possibly due to lack of immunity and adolescents were directly exposed to the mosquito vector (*Culex* sp.) bite, as they usually took active part in cultivation in crop-fields where vectors usually breed. In the present study, JE was found to occur in the monsoon period with the maximum number of cases in September when the *Culex* mosquitoes breed in the paddy fields covered with stagnant rain water.

However, we found that 86 [(99–61)+(61–13)] samples with a history of 2–15 days’ illness were true JE negative possibly due to either mishandling of samples which damaged the IgM antibody/the viral titre or the presence of another etiology responsible for AES.

In our previous reports we have achieved 36 JEV isolates [13] belonging to GIII whose E gene sequences were submitted to NCBI GenBank database like GenBank: JN189782, JN189783, JN189784 and HQ891146 etc. (unpublished data). The present study, therefore, constitutes the first report on E gene based phylogenetic analysis of the JEV isolates from AES cases of West Bengal where JEV GI has emerged very recently, co-circulating with JEV GIII.

Our study reveals that 2 isolates (GenBank: JN703381, JN703382) belonging to GI of JEV from the coastal district Midnapore (22.25°N 87.65°E and 23 meters above sea-level) where this GI might be transmitted from other part of India [7] or by the travellers returning from JE endemic countries, possibly with JEV infection [15]. On the other hand, this district has got many Lakes, swamps, forest and rice fields which provide a wintering and staging grounds for several migratory birds. Such areas are also very suitable for breeding and survival of mosquitoes. In view of these conditions, GI of JEV might have been introduced into West Bengal through migratory birds or cyclonic wind-blown mosquitoes from newer geographic region [16,17]. However, it is still unknown as to how JEV GI has emerged in West Bengal exactly. Therefore, further studies to determine the role of travelers, migratory birds and wind-blown mosquitoes in JEV transmission are required.

The State Health Department of Government of West Bengal undertook the vaccination programme against JE in Midnapore in 2008 [13] using live attenuated JE vaccine derived from GIII strain SA-14-14-2. In this connection, the efficacy of the vaccine to protect against GI of JEV needs careful evaluation. In near future, there is a chance for an impending threat of JEV outbreak in this region/state, if this vaccine fails to protect sufficiently against GI of JEV.

## Competing interests

The authors declare that they have no competing interests.

## Authors’ contribution

AS and SCHAT conceived the study, the design, and drafted the manuscript. AS and DT carried out serology and molecular work. AS, DT, SKM, SCHAK and SCHAT contributed to the data analysis and data interpretation. All authors read and approved the final manuscript.

## Ethical approval

The study was duly approved by the joint ethical committee of ICMR (Indian Council of Medical Research) virus unit and NICED (National Institute of Cholera and Enteric Diseases), Kolkata, India.
